# Role of the Mechanisms of Detection in the Increased Risk of Thyroid Cancer: A Retrospective Cohort Study in an HMO in Buenos Aires

**DOI:** 10.1155/2018/8986074

**Published:** 2018-07-15

**Authors:** María Fabiana Russo Picasso, Jimena Vicens, Carina Giuliani, Ana del Valle Jaén, Carmen Cabezón, Marcelo Figari, Ana María Gómez Saldaño, Silvana Figar

**Affiliations:** ^1^Department of Endocrinology, Metabolism and Nuclear Medicine, Hospital Italiano de Buenos Aires, Perón 4190 (1202), CABA, Argentina; ^2^Epidemiology Section of the Department of Medicine & Department of Research, Hospital Italiano de Buenos Aires, Perón 4190 (1202), CABA, Argentina; ^3^Head and Neck Unit of the Department of Surgery, Hospital Italiano de Buenos Aires, Perón 4190 (1202), CABA, Argentina; ^4^Pathology Department, Hospital Italiano de Buenos Aires, Perón 4190 (1202), CABA, Argentina; ^5^Epidemiology Section of the Department of Medicine, Hospital Italiano de Buenos Aires, Perón 4190 (1202), CABA, Argentina

## Abstract

**Background:**

Two hypotheses attempt to explain the increase of thyroid cancer (TC) incidence: overdetection by excessive diagnostic scrutiny and a true increase in new cases brought about by environmental factors. Changes in the mechanism of detection and the risk of incidentally diagnosed TC could result in an increase of TC incidence.

**Methods:**

Retrospective cohort study. We identified incident cases of TC from the pathological reports of patients in a HMO and review of clinical records. The results were analyzed in two periods: 2003-2007 and 2008-2012. Incidence rates expressed per 100,000 person-years (with 95% CI) and relative risk of incidence rates of incidental and nonincidental TC were estimated.

**Results:**

The relative risk of incidentally detecting a thyroid cancer in 2008-2012 compared to 2003-2007 was 6.06 (95%CI 1.84-20.04). Clinical evaluations detected 31 (75.6%) cancers in the period 2003-2007 and 70 (51.8%) cancers in the period 2008-2012 (p<0.007). Although tumor median size was significantly lower in the period 2008-2012 (10 vs. 14 mm, p<0.03), tumors greater than 40 mm (4.3%) were only present in 2008-2012. The female/male ratio decreased between analyzed periods from 8 (3-21) to 4 (3-7).

**Conclusions:**

Our findings partially support the hypothesis of increased incidence due to overdetection but do not explain the changes in the increase of larger tumors and decrease in the female/male ratio, which could be secondary to the influence of unidentified environmental factors.

## 1. Introduction

Thyroid cancer is the most common endocrine cancer and its incidence has increased worldwide in the past 20 years [1]. Two hypotheses attempt to explain this rapid increase: overdetection or a true increase of thyroid cancer. We prefer the term “overdetection” to “overdiagnosis” when referring to the detection of small, clinically irrelevant disease by excessive diagnostic scrutiny, as this better reflects an excess in detection and not an error in the diagnosis of thyroid cancer. Alternatively, a true increase in new cases brought about by environmental factors could result in an increase of thyroid cancer incidence. Identifying the mechanism behind the increase of thyroid cancer incidence is vital because it might influence the policies designed to control this important health issue.

Several observations favor the overdetection hypothesis, such as autopsy studies that show the existence of a reservoir of indolent thyroid cancer that is never manifested during the patients' lifetime [2] and the fact that most of the increase in incidence is attributable to papillary cancers of less than 1 cm [3]. The association between the incidence rates and the levels of access to medical care [4], the use of diagnostic procedures [5–7], and the unchanged mortality rate [3] further support this hypothesis. A recent report has estimated that overdetection accounted for 90% of thyroid cancers diagnosed in women in South Korea and 70–80% in the United States, France, Italy, and Australia from 2003 to 2007 [8]. On the other hand, some authors have found an increase in incidence of larger tumors, suggesting that overdetection is not the only explanation [9]. Moreover, the concept that mortality has remained unchanged has been challenged recently by authors reporting that the incidence-based mortality has, in fact, increased in thyroid cancers of all SEER stages at diagnosis, thus suggesting a real increase in the incidence of thyroid cancer [10]. The global increase in obesity and insulin resistance has also been implicated as a predisposing factor in the development of differentiated thyroid cancer [11–13], whereas other environmental factors, such as endocrine disruptors, xenobiotics, and even viruses, could promote the development of thyroid cancer acting through genetic or epigenetic mechanisms [14, 15].

Most researchers agree that overdetection refers to clinically asymptomatic disease that is detected by serendipity. However, the impact of the changes in the mechanism of detection on the increase in incidence has not been extensively studied. The most probable reason for this is that the mechanism of diagnosis is not available in population-based cancer registries, precluding the communication of incidence rates in terms of the mechanism of diagnosis or the presence of symptoms. Some authors have made significant contributions by assessing the triggers of detection in hospital-based populations [16] and at the county level [17]. In their population-based study, Brito et al. found that the increase in incidence of thyroid cancer in the 2000–2012 period was completely attributable to the detection of clinically occult lesions in ultrasound studies [17]. This suggests that the increase in incidence is more apparent than real and that public policies designed to curb this so-called epidemic should probably be aimed at the method of detection.

In Argentina, an increase in thyroid cancer incidence has been reported by Faure and Cohen-Sabban [18, 19], but there are no data about the mechanism of detection to help distinguish whether this increase can be ascribed to an increase in medical scrutiny, that is, overdetection. Thus, our aim in this study was to determine the differences in the mechanism of detection and the risk of incidentally diagnosed thyroid cancer over time in the members of the Hospital Italiano HMO at Ciudad Autónoma de Buenos Aires (CABA). We also assessed epidemiological characteristics of thyroid cancer in our cohort.

## 2. Materials and Methods

### 2.1. Design

It is a retrospective observational cohort study.

### 2.2. Setting

Argentina has three main healthcare providers: the public health service, social security associated with labor unions, and private health insurance. Sixty-five percent of the population is covered by social security and private insurance through 300 organizations of different sizes and levels of care [20].

Plan de Salud at Hospital Italiano (PSHI) is an HMO that covers the healthcare needs of a well-defined population of 160,000 members living in the City of Buenos Aires and its suburbs. It provides its services through two main medical centers and 24 outpatient clinics located in the city's suburbs. The last census, in 2010, reported that the total population living in the Buenos Aires metropolitan area was 2,890,151 people in Buenos Aires' inner city and 9,916,715 people in the suburbs [21].

The PSHI uses an in-house electronic health records (EHR) system that is the repository of all the information of the patients' healthcare interventions and includes medical notes, laboratory results, and diagnostic imaging studies. Patients are clearly identified in the database by name, healthcare provider, photograph, identification number, and date of birth to prevent record duplication or misallocation.

This study was performed in accordance with the tenets of the Declaration of Helsinki and approved by the Ethics Committee of the Hospital Italiano de Buenos Aires.

### 2.3. Study Population and Case Definition

The study population included male and female members of the PSHI who were 18 years of age and older and had active memberships at the time of their thyroid cancer diagnosis. We defined the incident cases as every patient with newly diagnosed thyroid cancer that underwent surgery, at either of the two PSHI medical centers between January 1, 2003, and December 31, 2012. To avoid an overestimation of the incidence rates by the inclusion of preexisting thyroid cancer cases, we excluded patients with less than a 12-month affiliation from our cohort. We also excluded cases of secondary involvement of the thyroid by other cancers, such as metastases of other malignancies in the thyroid or an invasion by cancers of the larynx or the esophagus.

### 2.4. Data Management

Data source and retrieval: Cases of thyroid carcinoma in PSHI patients were obtained from the Pathology Department's database. The query began by selecting all the thyroid gland pathology reports. The cases of malignant neoplasms were identified and validated by reviewing the clinical records of the patients. Validation included the confirmation of each case as incident and checking that the patients' membership was active the day the diagnosis of thyroid cancer was established.

Demographic and tumor characteristics were retrieved by accessing the electronic medical record of each patient by one of two investigators (CG, MFRP). Our variables of interest were as follows: sex; age at thyroid surgery; tumor characteristics in the pathology report; mechanism of detection; and clinical risk factors, such as family history of thyroid cancer and radiation exposure. We considered the date of the pathology report of the thyroid surgery as the incidence date. Tumors were staged according to the 7th edition of the American Joint Committee on Cancer (AJCC) [2009]. Concordance between investigators was maximized by a discussion of definitions and a coding of endpoints in investigator group sessions as well as by reviewing inconsistent cases by two of the investigators. Data entry error was minimized by checking the database for inconsistencies and missing data.

For each incident case, a mechanism of detection was assigned. Definitions for each category of detection are shown in Table 1. Briefly, we considered three main mechanisms of detection: incidental finding, self-detection when this occurred outside the healthcare system, and clinical evaluation of known thyroid disorders or findings during the physical examination. For comparison purposes, methods of detection were regrouped according to clinical presentation: tumors detected by either the attending physician or self-detected were considered clinically evident or nonincidentally detected. Cases without clear information about the method of detection were included in the description of the tumor characteristics and risk factors but not in the estimation of the incidence rates.

### 2.5. Statistical Analysis

Data were analyzed in two periods of time, 2003–2007 and 2008–2012, to enhance precision and to avoid the effect of interannual variability of the incidence rates expected due to a low number of cases. Clinical and tumor characteristics were described as follows: quantitative variables were summarized by a mean (standard deviation) or median (interquartile range), depending on their distribution, whereas categorical variables were expressed as an absolute frequency (and percentage). To compare the case characteristics between periods, we performed a* t*-test or a chi-squared test, depending on the type of variable. Mann–Whitney and Fisher tests were used when appropriate. The Bonferroni correction was used to adjust for multiple comparisons (estimated threshold 0.017 for 3x2 tables).

The cumulative rate for incidentally diagnosed and nonincidentally diagnosed tumors was estimated for each period as cases per 100,000 persons per period with their corresponding 95% confidence intervals. Rates were age-standardized by the direct method to the World Health Organization (WHO) standard population. To compare the risk of detecting incidentally and nonincidentally diagnosed tumors between periods, we estimated incidence rate ratios with their respective 95% confidence intervals. The results were calculated as the ratio between the cumulative incidence rate between the second and the first periods. Confidence intervals were estimated using the Poisson distribution.

## 3. Results

We retrieved a total of 189 cases of thyroid cancer patients belonging to the PSHI, who underwent surgery from 2003 to 2012. There were no differences of the age at presentation between periods. There was an overall preponderance of female patients (158; 83.6%) but a decrease in the female/male ratio from 2008 to 2012 compared to the 2003–2007 period, although this was not statistically significant (Table 2).

### 3.1. Tumor Characteristics

The histopathological diagnosis was available for all the patients. The most common type was papillary thyroid carcinoma (n = 166; 87.8%) followed by follicular thyroid carcinoma in 16 patients (8.4%). The distribution of differentiated thyroid cancer types (n = 182; 96.2%) did not differ between periods, nor did the prevalence of the different subtypes of papillary thyroid carcinomas although this analysis was restricted by small numbers (data not shown).

**Figure 1:**
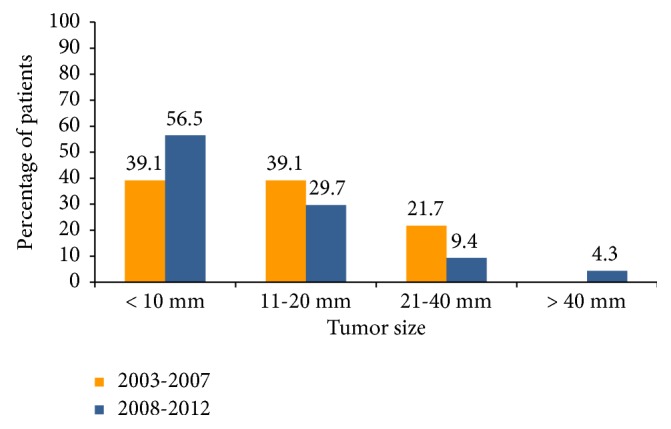
Tumor size categories by diagnostic period in members of the PSHI 2003-2012.

Size and thyroid capsule involvement was recorded in 184 pathology reports (97%). The tumor size was significantly larger in men than in women [median 15 mm (20) vs. 10 mm (9); p<0.02]. The tumor size was also significantly smaller in cancers operated on in the period 2008-2012, median 10 mm (10), when compared to those operated on in the period 2003-2007, median 14 mm (10), p<0.03. Coincidentally, microcarcinomas were more prevalent in that period as well (OR 2.02; 95% CI 0.97–4.26). Tumors greater than 4 cm were only present in the period from 2008 to 2012 (n=6), although this did not attain statistical significance (Figure 1). These large tumors were mainly classic, unencapsulated papillary thyroid cancers but all had either vascular invasion or extrathyroidal extension; one of them was an encapsulated solid variant papillary cancer and one was a follicular thyroid cancer. Of note, no follicular variants of papillary thyroid cancer were identified amongst the large thyroid cancers. When considering the whole cohort of thyroid cancers, extrathyroidal extension was present in 27 patients (14.7%), although it was mainly microscopic (21 patients, 11.4%). There was also a significant increase in extrathyroidal extension in patients operated on in the period from 2008 to 2012 (p <0.03) (Table 1).

### 3.2. Mechanism of Detection

Physical examination or diagnostic procedures of the thyroid identified 101 thyroid cancers between 2003 and 2012 (53.4%). Incidental findings by screening or studies aimed at other organs, including staging of other cancers, accounted for 44 (23.3%), whereas another 31 (16.4%) cancers were detected by the patient or someone outside the healthcare system. Thirteen patients had no information relating to the trigger of discovery in the medical notes (6.9%) and were excluded from the comparison between periods (definitions of methods of diagnosis in Table 1).

Seventy-one percent (n = 72) of thyroid cancers detected by clinical evaluation were discovered during the surveillance of thyroid dysfunction or benign thyroid nodules. Incidentally detected cancers were mainly discovered through imaging studies (n = 36; 81%), and of those 26 (72%) were detected by neck ultrasound studies in patients without a known thyroid disease or by carotid artery flow studies. Self-detected tumors were associated with compressive symptoms in seven patients (3.7%). Furthermore, the method of detection differed by gender. A clinical evaluation was the main trigger of detection in women [n = 92 (62%) vs. n = 9 (33%); p <0.006 for a Bonferroni threshold of 0.017], whereas incidentalomas were more frequent in men [11 (41%) vs. 33 (22%); n.s.].

**Figure 2:**
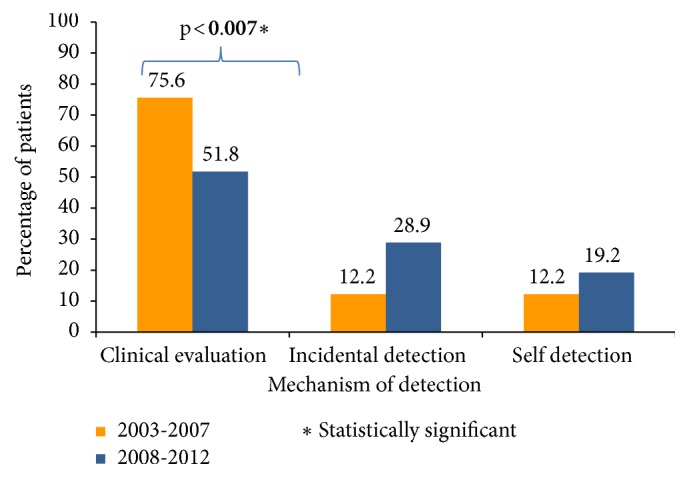
Mechanism of detection by diagnostic period in members of the PSHI 2003-2012.

There was also a significant change in the method of detection between the periods (p <0.024). Clinical evaluations detected 31 (75.6%) cancers from 2003 to 2007 and 70 (51.8%) cancers from 2008 to 2012 (p <0.007). Incidental detection accounted for 5 (12.2%) thyroid cancers in the first period and 39 (28.9%) in the second period (p <0.03). The proportion of self-detected thyroid cancer remained unchanged in the two periods [5 (12.2%) vs. 26 (19.2%); n.s.]. After adjusting for multiple comparisons, only the decrease in detection by clinical evaluation in the second period attained a statistical significance. However, 88% of all the incidentally detected tumors were discovered in the second period (Figure 2). Clinically evident tumors accounted for 83.7% (n = 36) of thyroid cancers diagnosed from 2003 to 2007 and were significantly reduced in prevalence from 2008 to 2012 (n = 96; 71.1%) (p <0.03).

### 3.3. Risk Analysis

The standardized cumulative incidence rate (SIR) of incidental thyroid cancer was 0.7 (95% CI: 0.14–1.96) from 2003 to 2007 and six (95% CI: 3.97–8.77) from 2008 to 2012. The SIRs for nonincidental thyroid cancer were six (95% CI: 3.97–8.77) and 10.6 (95% CI: 8.25–13.47), respectively (Table 3). The relative chance of incidentally detecting a thyroid cancer between 2008 and 2012 compared to 2003–2007 was 6.06 (95% CI: 1.84–20.04). The relative chance of detecting a thyroid cancer by self-diagnosis or clinical evaluation was 1.76 (95% CI: 1.13–2.75) when comparing the last period over the first period of the study. Crude and age-adjusted cumulative incidence for the incidental and nonincidental detection for each period is shown in Table 3.

## 4. Discussion

Thyroid cancer is the most common endocrine cancer, and its worldwide increase in incidence poses a major public health problem [1]. There is an ongoing controversy about whether this increase is real or apparent. The identification of the method of detection of these new cancers can contribute to the clarification of this issue. Our study found that in a well-defined cohort belonging to a private health insurance program, which serves a portion of the population of the city of Buenos Aires and its environs, the risk of incidentally detected thyroid cancer has increased over five times in the past few years, whereas the risk of nonincidentally detected thyroid cancer increased by only about 76%.

The characteristics of thyroid cancer in our cohort showed that it mainly involved middle-aged females. Likewise, most of the tumors were papillary thyroid cancers in low-risk stages of disease. These characteristics did not change between periods. On the other hand, the size was considerably smaller between 2008 and 2012. Our findings were in agreement with other reports in Argentina [18, 19], the region [22], and other countries [3],which have been interpreted to support the hypothesis of overdetection as the main reason behind the increase of thyroid cancer incidence. Furthermore, we showed that initial staging and multifocality did not change across time periods, suggesting that the most newly diagnosed thyroid cancers in our institution comprised tumors of low aggressiveness and possibly clinically inert ones.

In our study, we described the triggers of detection of newly diagnosed thyroid cancers as a means of exploring a possible explanation for the worldwide increase in thyroid cancer incidence. We found that the way that these tumors were detected has changed in recent years, with a significant decrease in the proportion of tumors detected during medical evaluation, that is, during a routine physical exam or in the workup of a benign thyroid disease. Conversely, thyroid cancers that were discovered incidentally during an imaging study increased in the same period. Although this increase did not attain statistical significance, probably due to the relatively few cases in our series, it is epidemiologically relevant because there was also a significant decrease in the proportion of clinically evident tumors in the second period.

Other authors have described triggers of detection albeit with different definitions than ours, which makes comparisons difficult. In their 2010 report, Davies et al. [16] found that 46% of cancers were detected during a routine physical exam or the surveillance of benign thyroid diseases, being lower than 59% of cases of our patients that were detected in the same way (Supplementary Materials, available here). However, their series had a remarkable number of symptomatic thyroid cancers (32%), probably reflecting the characteristics of their local practice. As put forward by A Van den Bruel et al., thyroid cancer incidence is closely related to the rate of thyroid surgery and is lower in the regions where surgery is not as frequently used to treat euthyroid nodular disease [6]. Davies and colleagues excluded cases of thyroid surgery used to manage endocrine disease or familial endocrine neoplasia and subsequently found only one surgical incidentaloma [16]. It is possible that their series was enriched by symptomatic cases at the expense of asymptomatic surgical incidentalomas by restrictions of surgery to symptomatic disease or abnormal FNA results in their local practice.

Our 4% of symptomatic thyroid cancers were more in accordance with a clinical setting and are closer to the 11% reported by Brito et al. at the county level [17]. More importantly, the two hospital-based reports concur in the proportion of thyroid cancers found by incidental imaging or the diagnostic cascade of unrelated symptoms: 22% in the Davies study and 19% in our study. Our series excluded patients with known benign thyroid diseases and benign nodules from the incidental imaging category, which are included in the Olmsted county series when unrelated to the presenting symptoms. This could account for the differences between both reports: 36% in the Olmsted report and 19% in ours. We further established the importance of this change in the method of detection by estimating the risk of incidentally diagnosing a thyroid cancer, which was significantly higher in the second period. This result is underscored by the fact that the relative risk of diagnosing thyroid cancer nonincidentally was much lower, thus reflecting a disproportionate increase of incidental tumors.

Overall, our results suggested that many of the newly diagnosed thyroid cancers were smaller and in early stages of disease and that there was an increase in incidental detection in the past few years, thus supporting the hypotheses of overdetection. There were, however, unexpected findings in our series that would seem to contradict this conclusion as the only explanation for the increase in incidence of thyroid cancer. There was a significant increase of extrathyroidal extension and tumors greater than 4 cm. were only present in 2008-2012. This group of large papillary cancers showed features associated with invasiveness or recurrence but numbers were too small to elucidate their significance. There was also a concurrent decrease in the female/male ratio in the second period. Elisei and coworkers found a decrease in the female/male ratio and an increase in the prevalence of microinvasion in their retrospective review of 4,187 patients followed over 35 years in a single institution in Pisa [23]. As with our cohort, they found an increase in diagnosis through imaging studies and attributed the increase in male tumors to the increase of carotid artery flow studies but, overall, the tumors detected from 1990 to 2004 in their study were smaller and showed less nodal and distant metastasis. Our cohort is more recent than the Elisei series, which suggests that the differences in results, such as the magnitude of change in the female-to-male ratio (approximately 50% in our study and 15% in theirs), could be explained not only by different patterns of disease, but also by trends of clinical practice in time. In our case, the increase in microinvasion could also reflect a change in the method of reporting thyroid surgical specimens, per the protocol of the American College of Pathologists [24].

There seems to be no clear explanation for the increase in larger tumors, which would purportedly be clinically evident. This pattern could be limited to the region where it was assessed; Chen et al. found that the incidence rates for larger and more advanced tumors also increased in the United States over the same period of time as the study by Elisei, who did not [25]. Kilfoy et al. also described an increase of larger tumors across time periods [26]. Furthermore, these authors found that the female/male rate ratio was significantly lower in larger tumors and at older ages at diagnosis. They did not, however, find differences in the female-to-male rate ratio in terms of the period of diagnosis. We found that tumors were larger and predominantly incidental in men, with a concurrent decrease in the female/male ratio in the second period; that is, the detection of thyroid cancer increased proportionally more in men than in women. This could be the result of (1) a change in clinical practice that has increased screening for thyroid abnormalities in men, (2) an increased use of imaging studies aimed at other neck organs (e.g., carotid artery flow studies) in men that serendipitously detects thyroid tumors, (3) physical exams of men not including routine neck palpation and therefore detection of thyroid cancers in men at larger sizes, (4) a yet-unidentified factor, possibly environmental, that stimulates the development of larger tumors in men. The small numbers in our cohort precluded us from further examining these hypotheses.

Taken together, all these characteristics could be the expression of a small increase of more aggressive tumors that could be attributed to other causes, such as environmental factors. There is no predominant theory of environmental effects on the development of differentiated thyroid carcinoma. Exposure to radiation in childhood has been extensively described as a predisposing factor as a result of both nuclear accidents and therapeutic use [27, 28], but the effects of exposure in adulthood are less clear [29]. Occupational exposure to ionizing radiation was also the main occupational hazard related to thyroid cancer in a review by Aschebrook-Kilfoy et al. [30]. However, a recent retrospective review of thyroid cancer pathological specimens described a decrease in RET/PTC rearrangements, thus downplaying the increase of radiation exposure as a driver of the increase in thyroid cancer incidence [31]. Iodine supplementation has also been proposed as a determining factor, mainly in the increase of papillary thyroid cancer and the prevalence of BRAF mutations [28]. This hypothesis is difficult to support in our environment, because iodine supplementation of salt was established by law in Argentina in 1962 and thus would not influence current incidence trends. Recent screenings of healthy euthyroid pregnant women living in CABA have confirmed iodine sufficiency in this population [32, 33]. The worldwide increase in obesity and its association with an increase in thyroid cancer have supported the notion of obesity as a risk factor for thyroid cancer [13]. This association has also been described in Argentina by Rezzonico et al. in a local cohort of similar characteristics and origin to ours. These authors found that insulin resistance expressed as a high HOMA-IR index was present in 50% of their group of thyroid cancer patients, and this association was stronger in obese patients [34]. The molecular mechanism pathways underlying this association are still obscure.

More recently, endocrine disruptors and xenobiotics have been proposed as oncogenic factors, but studies addressing this hypothesis in relation to thyroid cancer are scarce. The city of Buenos Aires has not been subjected to chemicals of volcanic fallout or nuclear accidents. However, endocrine disruptors can also be produced by industrial waste that contaminates the soil and water and enters the food chain. A recent epidemiological study in the region of Santa Fe, which is 300 km from CABA, found an increase in mortality caused by malignant tumors in three of the eight districts near the industrial belt surrounding the city of Rosario, but thyroid cancer was not involved [35]. A more recent survey in the region did not assess thyroid cancer incidence [36]. Other endocrine-disrupting chemicals such as widely distributed plasticizers like bisphenol A and phthalates and herbicides and pesticides can also alter endocrine function through genomic and epigenetic mechanisms altering the synthesis, transport, metabolism, and action of thyroid hormones [37]. Thyroid function relies on micronutrients such as iodide and selenium that can be altered by endocrine disruptors [38]. Glyphosate, which is widely used as an herbicide in the main soybean and corn-producing area surrounding the city of Buenos Aires, has been linked to disorders of selenium metabolism and could disrupt the synthesis of selenocysteine, which is a fundamental component of enzymes that protect the thyroid follicular cells from free radicals [37, 39]. Thyroid action at receptor level can also be compromised by the structural similarity of several endocrine-disrupting chemicals to thyroid hormones, such as polychlorinated and polybrominated biphenyls and bisphenol A [40]. Endocrine disruptors could also have indirect carcinogenic effects on the thyroid through their obesogenic, diabetogenic, and estrogen-like properties [37, 41]. However, most of the evidence of in vitro and animal studies has related endocrine disruptors to thyroid dysfunction, but to date there is no clear relationship to the increase of thyroid cancer incidence [37, 42]. Aschebrook-Kilfoy et al. explored the hypothesis of occupational exposure to pesticides and agricultural chemicals in their review of the literature on occupational hazards and thyroid cancer and found most results to be inconsistent [31]; only the Agricultural Health study by Freeman et al. found an increased risk of thyroid cancer in the highest quartile of atrazine exposure when compared to the lowest quartile [43].

Finally, the hypothesis of overdetection has been predicated mainly on the evidence of unchanged mortality across time periods [3]. This view has been recently challenged by a study by Hyeyeum et al. These authors have used the novel approach of estimating incidence-based mortality with the data from the SEER database linked to information from death certificates, which allows the estimation of mortality according to characteristics of the tumor at presentation. In their study, there was a significant overall increase of incidence and mortality of thyroid cancer, attributed to advanced-stage papillary thyroid cancer [10].

Our study has several strengths, the main one being that an expert multidisciplinary team validated the cases by accessing and reviewing medical records, thus ensuring that misallocation was minimized. The principal investigators exercised stringent control of data entry error, and more than 90% of the records were complete for all the variables we studied. Another strength is the strict inclusion criteria used in the calculation of incidence rates to avoid overestimation by prevalent cases, whereby we excluded patients who had fewer than 12 months' affiliation with the health plan.

The main caveat of our study is that the exposed population belongs to a private health insurance plan and is not geographically defined. It is therefore difficult to extend our results to the general population. Another limitation is the change in the registration system of the Pathology Department that could have resulted in a higher retrieval of cases in the second period. This would have compromised the validity of the results only if the change was different for incidental and nonincidental tumors. However, there is a role for epidemiological studies in well-defined populations belonging to healthcare programs, as a source of high-quality data that is otherwise inaccessible. As has been stated by other authors, access to medical records in a well-defined population allows the analytical evaluation of tumor characteristics and prognostic factors in relation to incidence rates that are not available to cancer registries [18].

## 5. Conclusions

We observed an increase in incidentally and nonincidentally detected thyroid cancers in the population belonging to a private healthcare plan of Buenos Aires, compounded by a much higher relative risk of incidental than nonincidental tumors. The changes in the methods of detection in our study support the hypothesis of overdetection of small tumors. However, there was a trend of increasing large tumors and tumors in men, which suggests that other mechanisms could be involved. More studies are needed to address possible reasons for these findings. Finally, considering the paucity of population-based cancer registries in Argentina, this study provides additional information about the epidemiology of thyroid cancer in our country.

## Figures and Tables

**Table 1:** Definitions of mechanisms of detection of thyroid cancer.

MECHANISM	DEFINITION
Incidental detection	Unexpected finding of thyroid cancer in an asymptomatic patient during a routine examination, imaging study, or surgical procedure, or a pathological examination of a surgical specimen in a patient with no thyroid mass, no benign thyroid disease, and no family history of thyroid cancer including ultrasounds performed during a multiphase check-up module.
Self-detection	Detection of thyroid cancer by the patient or people outside the healthcare system. This category includes patients with and without symptoms related to the nodule that harbored thyroid cancer.
Medical evaluation	Clinical evaluation with a presumptive diagnosis of thyroid cancer including a physical exam or diagnostic workup of a cervical mass or lymph node detected by a physician belonging to the healthcare system. This category also included findings by a diagnostic cascade for benign thyroid disease.

**Table 2:** Patient and tumor characteristics of incident cases of thyroid cancer in PSHI (2003-2012).

**Characteristic**		**2003-2007** **(n=47)**	**2008-2012** ** (n=142)**	**p-value & association measures**
Age at diagnosis in years, mean (SD)		57.2 (15.3)	57.4 (14.4)	0.94 _ _^a^
Female, n (%)		42 (89.4)	116 (81.7)	0.21^a^
F/M ratio (95% CI)		8.4 (3.3-21.2)	4.5 (2.9-6.8)	1.9 (0.7-6.7)^b^

Histological type, n (%)	Papillary	39 (83)	127 (89.4)	
	Follicular	6 (12.8)	10 (7)	
	Medullary	0	2 (1.4)	
	Anaplastic	0	1 (0.7)	
	Other	2 (4.2)	2 (1.4)	

TNM Stage, n (%)	I	33 (73.3)	104 (74.8)	0.24^a^
	II	5 (11.1)	5 (3.6)	
	III	3 (6.7)	15 (10.8)	
	IV	4 (8.9)	15 (10.8)	

Multifocal, n (%)		10 (21.3)	31 (22.1)	0.9^a^
Extra thyroidal extension, n (%)		2 (4.6)	25 (17.9)	0.03^a^

^a^Chi square.

^b^OR (95% CI).

**Table 3:** Risk analysis of incidentally and non-incidentally detected thyroid cancer between periods of study in members of the PSHI (2003-2012).

	Incidentally detected thyroid cancer		Non-incidentally detected thyroid cancer	
Period of study	Crude cumulative incidence^a^ (95%CI)	Age standardized cumulative incidence^a^ (95% CI)	Crude cumulative incidence^a^ (95%CI)	Age standardized cumulative incidence^a^ (95% CI)

2003-2007	1.1 (0.36-2.61)	0.7 (0.14-1.96)	8 (5.63-11.1)	6 (3.97-8.77)
2008-2012	6.1 (4.33-8.33)	4.1 (2.65-5.95)	15 (12.2-18.3)	10.6 (8.25-13.5)

Relative risk (95% CI)		6.06 (1.84-20)		1.76 (1.13-2.75)

^a^Per 100,000 affiliates.

## Data Availability

The crude data and computing codes used to support the findings of this study are restricted by the Comité de Etica de Protocolos de Investigación (CEPI) of the Hospital Italiano de Buenos Aires in order to protect patient privacy. These may be released upon application to the CEPI, who can be contacted through the corresponding author.

## Supplementary Materials

Supplementary Materials Table describing distribution of patients in categories and subcategories of methods of diagnosis of thyroid cancer.
