# Supplementary material for: Role of the Mechanisms of Detection in the Increased Risk of Thyroid Cancer: A Retrospective Cohort Study in an HMO in Buenos Aires
Source: J Cancer Epidemiol. 2018 Jul 15;2018:8986074. doi: 10.1155/2018/8986074 (PMC6076902; doi:10.1155/2018/8986074)
Supplement: Supplementary Materials — Table describing distribution of patients in categories and subcategories of methods of diagnosis of thyroid cancer. [file 8986074.f1.pdf]

Supplementary Table 1 Types and subtypes of mechanisms of detection of thyroid cancer in PSHI 2003-2012

MECHANISM OF DETECTION		N	%
Clinical evaluation			
	Clinically evident thyroid mass	26	25.7
	Neck lymphadenopathy	1	0.9
	Preexistent benign thyroid disease	72	71.3
	Screening in high risk groups	2	1.9
	Total	101	100
Incidental detection			
	Imaging studies	36	82
	Thyroid ultrasound	12	
	Neck ultrasound	7	
	Carotid Artery Doppler	7	
	CAT Scan o MRI	10	
	Other	2	
	Surgery	8	18
	Total	44	100
Self detection			
	Visible or palpable mass	24	77
	Compression symptoms	7	23
	Total	31	100
